# Requirements for Unobtrusive Monitoring to Support Home-Based Dementia Care: Qualitative Study Among Formal and Informal Caregivers

**DOI:** 10.2196/26875

**Published:** 2021-04-12

**Authors:** Christian Wrede, Annemarie Braakman-Jansen, Lisette van Gemert-Pijnen

**Affiliations:** 1 Centre for eHealth and Wellbeing Research Department of Psychology, Health & Technology University of Twente Enschede Netherlands

**Keywords:** in-home monitoring, ambient assisted living, assistive technologies, dementia, home care, informal care, aging in place

## Abstract

**Background:**

Due to a growing shortage in residential care, people with dementia will increasingly be encouraged to live at home for longer. Although people with dementia prefer extended independent living, this also puts more pressure on both their informal and formal care networks. To support (in)formal caregivers of people with dementia, there is growing interest in unobtrusive contactless in-home monitoring technologies that allow caregivers to remotely monitor the lifestyle, health, and safety of their care recipients. Despite their potential, these solutions will only be viable if they meet the expectations and needs of formal and informal caregivers of people with dementia.

**Objective:**

The objective of this study was to explore the expected benefits, barriers, needs, and requirements toward unobtrusive in-home monitoring from the perspective of formal and informal caregivers of community-dwelling people with dementia.

**Methods:**

A combination of semistructured interviews and focus groups was used to collect data among informal (n=19) and formal (n=16) caregivers of people with dementia. Both sets of participants were presented with examples of unobtrusive in-home monitoring followed by questions addressing expected benefits, barriers, and needs. Relevant in-home monitoring goals were identified using a previously developed topic list. Interviews and focus groups were transcribed and inductively analyzed. Requirements for unobtrusive in-home monitoring were elicited based on the procedure of van Velsen and Bergvall-Kåreborn.

**Results:**

Formal and informal caregivers saw unobtrusive in-home monitoring as a support tool that should particularly be used to monitor (the risk of) falls, day and night rhythm, personal hygiene, nocturnal restlessness, and eating and drinking behavior. Generally, (in)formal caregivers reported cross-checking self-care information, extended independent living, objective communication, prevention and proactive measures, emotional reassurance, and personalized and optimized care as the key benefits of unobtrusive in-home monitoring. Main concerns centered around privacy, information overload, and ethical concerns related to dehumanizing care. Furthermore, 16 requirements for unobtrusive in-home monitoring were generated that specified desired functions, how the technology should communicate with the user, which services surrounding the technology were seen as needed, and how the technology should be integrated into the existing work context.

**Conclusions:**

Despite the presence of barriers, formal and informal caregivers of people with dementia generally saw value in unobtrusive in-home monitoring, and felt that these systems could contribute to a shift from reactive to more proactive and less obtrusive care. However, the full potential of unobtrusive in-home monitoring can only unfold if relevant concerns are considered. Our requirements can inform the development of more acceptable and goal-directed in-home monitoring technologies to support home-based dementia care.

## Introduction

### Background

Dementia is recognized as a major global health challenge, creating an immense rise in demand for care [1]. In 2019, the number of people with dementia worldwide was estimated at 50 million, a figure set to increase to 152 million by 2050 [2]. However, the quantity of available professional caregivers is not expected to increase along with the growing demand from an aging society [3]. Consequently, people with dementia will increasingly be encouraged, when possible, to live at home for longer [1]. Although extended independent living is preferred by people with dementia [4], this places more pressure on their informal and formal support network [5]. Most care for people with dementia is provided by unpaid informal caregivers such as spouses or relatives [6] who can feel heavily burdened by their care responsibilities, often resulting in stress-related illnesses [5,7], putting them at risk of becoming the so-called “invisible second patient.” On the other side, formal caregivers involved in the home care of people with dementia often face an increased workload due to a rising shortage of staff [8,9] and the growing complexity of care [10], which require them to use their resources more effectively [11].

To support caregivers of people with dementia in home-based settings, there has been growing interest in assistive technologies for in-home monitoring. These surveillance systems provide 24/7 information about the daily functioning, lifestyle, and safety of people with dementia, possibly leading to a greater sense of control, which could help to delay the institutionalization of this population [10,12,13]. The development of in-home monitoring systems is progressing and is increasingly driven by the aim to minimize their obtrusiveness; that is, to reduce ‘’characteristics or effects associated with the technology that are perceived as undesirable and physically and/or psychologically prominent’’ [14]. In practice, this is visible by moving away from in-home monitoring systems based on cameras, which pose major challenges concerning privacy [15,16]. Although more recent systems based on wearable sensors are less obtrusive when it comes to privacy, they are often more obtrusive in terms of the inconvenience associated with having to wear the system on the body [15,16]. To overcome these barriers, more unobtrusive, contactless in-home monitoring systems have been developed. These range from event-based, mostly motion-activated, sensors distributed in the home [10,17,18] to the most novel form of unobtrusive in-home monitoring systems based on analyzing the human body’s reflection of radio waves using deep-learning algorithms and artificial intelligence (AI) [15,16,19,20]. The continuous monitoring of in-home activity through these AI-driven systems could help caregivers to detect small but meaningful changes over time, monitor disease progression [21], and not only detect but also predict and subsequently prevent falls [22].

Despite the promise of the possibilities of unobtrusive in-home monitoring, the added value and downside for informal and formal caregivers of people with dementia are still insufficiently mapped out. Research on unobtrusive monitoring systems that could be applied to support home-based dementia care has largely focused on its technical possibilities [21-24] instead of investigating what is needed from the technology to support caregivers of people with dementia in an unobtrusive and goal-directed manner. A recent review by Vermeer et al [13] highlights the importance of reporting concrete requirements for monitoring systems in dementia care so that they can be used by technology developers and based on the perspective of those who might use them. However, previous research on unobtrusive in-home monitoring involving (potential) end users [25-28] often failed to take these aspects into account, and mainly included the views of healthy older adults and their family members, with less attention devoted to the perspective of formal and informal caregivers of people with dementia who can be considered important target users. Furthermore, as unobtrusive in-home monitoring systems develop rapidly, previous studies involving end users are likely to become quickly out of date [13]. Advances in affective computing and AI are likely to add new possibilities such as monitoring emotion [29] or vital signs [30] remotely. At the same time, the shift to unobtrusive remote monitoring, which continuously creates and automatically models in-home data about people with dementia, might also present a new extent of threats related to privacy and ethics [18,31].

When developing meaningful, novel, and unobtrusive in-home monitoring technology with the goal of supporting home-based dementia care, an adequate comprehension of users’ needs is essential to reach a fit among technology, context, and the user [32]. Unobtrusive in-home monitoring of people with dementia should not be developed simply because it can be, but with its possible benefits and barriers in mind as well as with consideration of the relevant needs and requirements to increase future acceptance.

### Aim of the Study

Based on this background, the aim of this study was to comprehensively explore the views of formal and informal caregivers of community-dwelling people with dementia toward unobtrusive in-home monitoring. In particular, the study aims were to identify (1) relevant and nonrelevant monitoring goals of unobtrusive in-home monitoring, (2) expected benefits and barriers toward unobtrusive in-home monitoring, and (3) specific requirements for unobtrusive in-home monitoring technology that can serve as guidelines for developers.

## Methods

### Study Design

A qualitative research design was applied, including semistructured interviews and focus groups. Moreover, a topic list task was part of the interview and focus group sessions. The Ethics Committee of the University of Twente (Behavioral, Management, and Social Sciences) provided ethical approval for this study according to European regulations (request number 18939).

### Participants and Sampling Procedure

#### Informal Caregivers

Inclusion criteria for participation of informal caregivers were as follows: (1) providing unpaid care to a community-dwelling person diagnosed with dementia or mild cognitive impairment (MCI) at the time of data collection, and (2) providing care from a distance or living together with the care recipient. Recruitment took place during public information meetings at Alzheimer and informal care cafes organized by different Dutch elderly and informal care institutions. Those who met the inclusion criteria and were interested in participating were asked to provide their contact details and were given a detailed information leaflet introducing the study. After 3 days, participants were called and if they still wished to participate, an appointment for a home visit was made. In total, 19 informal caregivers were interviewed.

#### Formal Caregivers

Inclusion criteria for participation of formal caregivers required participants to be home care professionals that, at the time of data collection, provided and/or managed the care for community-dwelling people diagnosed with dementia or MCI. Recruitment took place from a variety of Dutch home care institutions and public Alzheimer cafes where formal caregivers were informed about the purpose and procedure of the study. In total, 16 formal caregivers from 7 different home care institutions agreed to participate and were included in the study. Five caregivers were interviewed individually, followed by two focus groups (n=6 and n=5, respectively), which were held at two different local elderly care institutions.

### Topic List Task

During the interviews and focus groups, all participants performed a topic list task to identify relevant and nonrelevant monitoring goals for unobtrusive in-home monitoring. Each participant received 16 topics representing different possible goals of monitoring, covering key aspects of daily functioning, safety, degeneration, and well-being of people with dementia. These topics were previously developed in cocreation with our expert panel consisting of experienced geriatricians and gerontologists, and based on commonly used scales to assess activities of daily living [33,34] and stages of dementia [35], as well as research into (the course of) behavioral and psychological symptoms of dementia [36,37]. We also considered the type of input unobtrusive in-home sensors would require. The final list of goals was clustered into four broad areas of attention commonly used by formal caregivers across the continuum of care for individuals [38]. Participants were asked to rate each goal with either a plus sign (+), indicating that this would be a relevant monitoring goal, a minus sign (–), indicating a nonrelevant monitoring goal, or a question mark (?) indicating uncertainty about the usefulness of a monitoring goal. We instructed participants to envisage that all goals could technically be monitored to any useful level of precision. During the task, participants were encouraged to support their choices and add goals when possible.

### Interview and Focus Group Guide

We developed an interview and focus group guide containing open-ended questions based on our research questions, previous research in the field by Wild et al [25], and the value proposition design proposed by Osterwalder et al [39]. For both groups of participants, questions and procedures were essentially identical, except that the questions were adapted to fit the different roles and care duties of the groups. At the beginning of each interview and focus group, participants viewed two slides demonstrating the general concept and idea of unobtrusive in-home monitoring and examples to illustrate possible forms of outgoing monitoring information. All slides were explained in a standardized manner (see Multimedia Appendix 1) and clarification was given when needed. Subsequently, participants were asked about their perceived expected benefits of unobtrusive in-home monitoring. Questions targeting benefits mainly centered around why, if at all, participants would be willing to use such a system for their care recipient(s); how, if at all, it would influence the care and quality of care; and why or why not it would be able to support extended independent living of people with dementia.

Thereafter, participants performed the topic list task, followed by the last section that focused on concerns. Questions about concerns mainly centered around reasons not to use unobtrusive in-home monitoring, when such a system would become undesirable, and concerns related to their own and their care recipients’ privacy and data sharing. The interviews were performed by a trained interviewer (CW), and focus groups were led by a moderator (CW) and comoderator (AB). After 24 interviews and 2 focus groups, saturation was reached. All sessions were audio-recorded, with prior permission of participants (see Multimedia Appendix 2 for the full interview and focus group guide). Interviews lasted about 1 hour and focus groups lasted about 1.5 hours each.

### Data Analysis

The audiotapes of the interviews and focus groups were transcribed verbatim and content analysis was performed using the software package Atlas.ti 8 separately for each participant group. First, relevant fragments were selected and categorized into one of two main areas: (1) expected benefits and (2) expected barriers toward unobtrusive in-home monitoring. Subsequently, selected fragments were further categorized inductively into overarching themes and subthemes. To minimize single-researcher bias, a second analyst (AB) independently coded 10% of the data and verified whether the codes described were proper interpretations of the data. The final coding scheme was defined on the basis of consensus between the two analysts (CW and AB).

Data from the topic list of monitoring goals were used to generate frequency distributions across all response categories (relevant, nonrelevant, or questionable monitoring goals) per participant group.

As a last step, requirements toward unobtrusive in-home monitoring were created based on the procedures of van Velsen et al [40] and Bergvall-Kåreborn and Ståhlbröst [41].

First, user expressions that captured aspects the system should fulfill were identified and clustered into overarching *attributes* (ie, needs that aim to guide the development). These were checked for distinctiveness by the second analyst (AB) and adjusted if needed.

Second, the *attributes* were translated into one or more *requirements* and categorized into four different domains: (1) functional requirements specifying desired technical features of the technology, (2) user experience requirements specifying how the technology should interact/communicate with the user, (3) service requirements specifying desired services surrounding the technology, and (4) work context requirements specifying how the technology should be integrated into the existing work context and routines. All requirements were checked by the second analyst (AB) and adjustments were made accordingly.

Lastly, all requirements were sorted by the corresponding overarching theme.

## Results

### Participant Characteristics

The characteristics of study participants are listed in Tables 1 and 2. Informal caregivers were children, partners, sisters- or brothers-in-law, or neighbors who provided care, from a distance or not, to a community-dwelling person with dementia or MCI. Most informal caregivers were daughters and sons providing care from a distance to their parent with Alzheimer disease. Notably, most informal caregivers reported being active as a caregiver for the patient several years before an official diagnosis was made. Formal caregivers were experienced home care professionals, including district nurses, case managers, care assistants, and occupational therapists providing on average of 20.3 hours (SD 6.3) of care per week to community-dwelling clients with dementia.

**Table 1:** Characteristics of informal caregivers (N=19).

Characteristic			Value
Age of caregiver (years), mean (SD)		60.5 (13.1)	
Age of care recipient (years), mean (SD)		82.6 (5.5)	
Active as caregiver (years), mean (SD)		10.5 (10.1)	
Time since diagnosis^a^ (years), mean (SD)		4.6 (3.5)	
**Gender, n (%)**			
	Female	16 (84)	
	Male	3 (16)	
**Care hours per week, n (%)^b^**			
	<8	6 (32)	
	8-24	7 (37)	
	24-40	6 (32)	
**Relation with care recipient, n (%)**			
	Daughter/son	11 (58)	
	Spouse/partner	5 (26)	
	Sister-/brother-in-law	2 (11)	
	Neighbor	1 (5)	
**Living situation, n (%)**			
	Living together with care recipient	7 (37)	
	Living elsewhere	12 (63)	
**Type of cognitive impairment of care recipient, n (%)**			
	Alzheimer disease	14 (74)	
	Lewy body dementia	1 (5)	
	Vascular dementia	1 (5)	
	Mild cognitive impairment	3 (16)	

^a^Time since diagnosis was self-reported by the informal caregiver.

^b^Percentages may not total 100 due to rounding.

**Table 2:** Characteristics of formal caregivers (N=16).

Characteristic			Value
Age (years), mean (SD)		39.3 (11.3)	
Care contact hours per week provided to community-dwelling people with dementia, mean (SD)		20.3 (6.3)	
Work experience in current home care profession (years), mean (SD)		15.4 (9.1)	
**Gender, n (%)**			
	Female	15 (94)	
	Male	1 (6)	
**Function^a^, n (%)^b^**			
	District nurse	8 (50)	
	Case manager for dementia	4 (25)	
	Personal care assistant	2 (13)	
	Occupational therapist	2 (13)	

^a^Secondary vocational education (European Qualifications Framework [EQF] level 1-4): personal care assistant, district nurse; higher professional education (bachelor/associate degree; EQF level 5/6): district nurse, case manager for dementia, occupational therapist.

^b^Percentages may not total 100 due to rounding.

### Goals of Unobtrusive In-Home Monitoring According to (In)formal Caregivers

Table 3 summarizes the responses from formal and informal caregivers toward possible monitoring goals of unobtrusive in-home monitoring systems that were defined during the topic list task. The top 5 monitoring goals seen as most useful by both groups included fall detection and prevention, and monitoring day and night rhythm, personal hygiene (eg, dressing, grooming, bathing, and toileting), nocturnal restlessness, and eating and drinking behavior. Monitoring goals rated as nonrelevant by both formal and informal caregivers included leaving the house (the action of doing so), social interaction (indoors), and telephone use (frequency of use). In general, both groups showed comparable results, although informal caregivers appeared to be less interested in monitoring eating and drinking behavior, nocturnal restlessness, and walking distance/speed compared with formal caregivers.

**Table 3:** Judgments of informal and formal caregivers of community-dwelling people with dementia for specific unobtrusive in-home monitoring goals.

Monitoring goals			Informal caregivers (N=19), n						Formal caregivers (N=16), n				
		+^a^		–^b^	?^c^		+			–		?	
Environmental (safety): fall detection and prevention		19		0	0		16			0		0	
**Health-related**													
	Day and night rhythm/sleeping pattern	18		1	0		15			1		0	
	Nocturnal restlessness	12		5	2		16			0		0	
	Personal hygiene (dressing, grooming, bathing, toileting)	15		2	2		15			1		0	
	Eating/drinking and cooking activity	12		7	0		16			0		0	
**Physiological**													
	Cognitive deterioration	13		5	1		15			1		0	
	Physical deterioration	10		7	2		12			2		2	
	Walking distance/speed (indoors)	10		6	3		14			1		1	
	Radius of movement (indoors)	14		5	0		8			4		4	
**Psychosocial**													
	Agitation (agitated behavior)	13		4	1		12			4		0	
	Apathy (lethargy/loss of motivation and interest)	12		5	2		9			4		3	
	Negative emotional state (eg, anxiety, irritability, depression)	12		6	1		11			4		1	
	Positive emotional state (eg, joy, pleasure, relaxation)	10		7	2		9			5		2	
	Leaving the house (the action of leaving)	5		7	7		6			6		4	
	Telephone use (frequency of use)	4		5	10		5			7		4	
	Social interaction (indoors)	4		11	4		7			6		3	
Total		183		83	37		186			46		24	

^a^Personally relevant.

^b^Not relevant.

^c^Questions the usefulness.

### Expected Benefits and Barriers Toward Unobtrusive In-Home Monitoring

#### Expected Benefits

##### Overview

Textbox 1 shows the recurrent themes on benefits of unobtrusive in-home monitoring that emerged for the informal and formal caregiver groups. The themes of both groups were generally in line with each other, with variations within these themes reflecting the different roles and care responsibilities of the groups.

**Expected benefits toward unobtrusive in-home monitoring stated by informal and formal caregivers of people with dementia.**

**Themes brought forward by both informal and formal caregivers**

*Cross-checking self-care information*

- Better self-care surveillance
- In-person control visits

*Extended independent living*

- Safety at home
- Detecting and removing factors that hinder independence
- Helpful for initiating extra care needed
- Decision support for transition to residential care

*Objective communication and substantiation*

- Supporting objective communication around patient’s situation
- Substantiating diagnostics and indications

*Prevention and proactive measures*

- Responding more quickly to care needs to prevent health risks
- Improved insight into inhibiting and activating factors of patient’s behavior/ mood

**Theme brought forward by only informal caregivers**

*Emotional reassurance*

- Reassurance about safety of patient
- Regain of freedom and mobility for informal caregiver

**Theme brought forward by only formal caregivers**

*Personalized and optimized care*

- Providing care at the right times
- Time gain through remote surveillance of self-care behaviors

##### Cross-Checking Self-Care Information

Informal and formal caregivers reported facing difficulties in obtaining complete information about the patient’s living pattern as the patient might not always be able to accurately self-report on the past few days. Participants indicated that, particularly in the prediagnostic phase or in cases of little or no home care provision, there is a lot of doubt about how adequately self-care practices such as eating/drinking and personal hygiene are performed. The monitoring system could then help in cross-checking what the patient self-reports:

I would find it very helpful if you could find out if they had eaten because they often told us they had when they hadn’t. [...] I saw with my parents that I didn’t notice anything when I came by, and if we could have followed this we could have intervened more quickly.

caregiving daughter, age 56

Therefore, according to participants, unobtrusive in-home monitoring can be a substitute for constantly having to (physically) check self-care behaviors. By eliminating unnecessary control visits, such systems might reduce the burden of care while supporting the patient’s physical privacy:

I think that you would have to visit someone less often, we regularly visit people just for controlling.

district nurse

##### Extended Independent Living

Informal and formal caregivers mainly expected more reassurance and safety with the use of unobtrusive in-home monitoring, which could indirectly contribute to patients being able to live at home for longer. Both groups saw potential in using such a system for detecting and removing factors or negative stimuli in the environment causing a patient, for example, to wander at night, which can ultimately hinder independent living:

People with dementia often have misunderstood behavior [...] Suppose there is a stimulus that causes someone to have nighttime unrest or to wander. Normally, wandering is a criterion that prevents someone from living at home. Suppose you can remove this stimulus because you know where it comes from, then you ensure that someone can stay home longer. And in that sense, I think the system is an added value.

case manager

Furthermore, both groups expected an unobtrusive in-home monitoring system to be helpful in initiating extra care, when needed, to prevent the collapse of day structure and routines. Moreover, participants noted that such a system could assist in determining to what extent living at home would still be reasonable:

I think it will help a lot to be able to say okay living at home is no longer responsible [...] When you have data for it, you can also look at a situation objectively, without adding emotion, because there are facts, and that might make the decision later that it is no longer possible at home easier.

caregiving daughter, age 42

Some participants noted that patients sometimes reside at home longer than is appropriate. Early detection of health risks by an in-home monitoring system could then enable a faster indication for admission to residential care. Formal caregivers emphasized that such a system could also bridge the time a patient is on the waiting list for admission.

##### Objective Communication and Substantiation

Informal caregivers, in particular, expected unobtrusive in-home monitoring to be helpful in providing others with an objective insight into how their loved one functions. Such a system might help the caregivers to be taken seriously when making their assessments, especially considering that people with dementia can often present themselves well in the company of others or during care times. Informal caregivers noted having difficulties in objectively explaining the daily challenges at home to others; therefore, the outgoing monitoring information/graphs would speak for themselves:

The caregiver is not believed [...] And if you then have very good friends who also say “Well, everything is not too bad, look, he gives such good answers.” [...] And then I say “Yes, but you have to experience it once for 24 hours.” Something like this [the system] would be fantastic, that you could show that.

caregiving wife, age 72

Formal caregivers mainly saw an added value in the objective, continuous manner of measuring when using an unobtrusive in-home monitoring system. Several formal caregivers reported that during care moments they often see snapshots of socially desirable behavior and that information from informal caregivers is not always reliable. Furthermore, they expressed that such a system could be an aid to substantiate diagnostics and indications:

Cognitive decline, well, that is super of course if you can monitor that instead of just taking the MMSE [Mini Mental State Examination].

district nurse

##### Prevention and Proactive Measures

From the interviews and focus groups, it became clear that informal and formal caregivers expected unobtrusive in-home monitoring systems to enable them to respond more quickly and accurately to care needs to prevent health risks such as malnutrition, under/overstimulation, sleep problems, and loneliness.

I think such as system is a good plan. Before you realize that something is wrong, you are 4 months further. If you understand something like this with sensors, you can intervene much sooner.

caregiving daughter, age 56

Similarly, in response to the question “What are further preventive measures that could be supported by the system?”, district nurses and occupational therapists indicated “stimulating for instance eating and drinking,” “meaningful daytime activities,” and “medication, and indeed, keeping people busy.” Another added, “it is also a bit of well-being that you can nicely link to this, care is very important but so is well-being, and preventing loneliness.”

Both groups also felt that an unobtrusive in-home monitoring system would improve their ability to recognize influencing factors on the patient’s behavior and mood, and enable responding in a proactive manner. Generally, participants felt that such a system could help to monitor what relaxes or irritates a patient to be able to proactively take action sooner, as well as to fall back on this knowledge at a later stage of dementia.

Well, you could measure, when the home care has washed her, is she sad afterward? […] And can you do something with it? Yes, then you can start to question: Is it because of the way it was done?

caregiving brother-in-law, age 45

##### Emotional Reassurance

Informal caregivers reported seeing added value in unobtrusive in-home monitoring in terms of emotional support and reassurance. In particular, wearable alarm systems have been criticized for providing little reassurance because patients often forget to wear them or because alarm buttons are not always pressed when they should be. Furthermore, informal caregivers highlighted that the insight into daily activities obtained through unobtrusive in-home monitoring can result in a sense of involvement at a distance, which can enable them to regain freedom and mobility to some degree:

[...] So if the system can indicate like everything is all right, and it is reliable, it also gives you a reassuring feeling. Just as I knew my brother was a caregiver a few years ago. Then I was reassured on a distance, you can also compare it to that.

caregiving daughter, age 50

For informal caregivers, it would be a great relief because they can leave for a while and the system takes it over.

caregiving wife, age 72

##### Personalized and Optimized Care

Formal caregivers expected that unobtrusive in-home monitoring would help them to work in a more person-centered way by tailoring care moments to the individual rhythm of the patient. For example, if a client gets out of bed at a deviating time, then a care moment can be scheduled accordingly:

With something like this [the system], it would be possible to provide care differently. If you see someone sleeps longer you could consider putting on those compression stockings a little later than originally planned.

district nurse

Specifically, in the prediagnostic phase, there can be doubt about self-care behaviors, causing formal caregivers to sometimes lose a substantial amount of time to physically control activities (eg, eating, drinking, and sleeping behaviors). In some cases, formal caregivers arrive and find that the patient is already asleep. Formal caregivers generally agreed that an unobtrusive in-home monitoring system that enables them to better supervise these factors can save them time:

[...] Sometimes we come for a check-up moment and then the client is asleep and at another moment he might be out of bed, wandering around. So you could take more targeted action instead of those check-ups, and see if you can go there at that moment.

district nurse

#### Expected Barriers

##### Overview

Textbox 2 summarizes the themes and subthemes brought forward on the expected barriers, which were consistent between the informal and formal caregivers.

**Expected barriers toward unobtrusive in-home monitoring brought forward by informal and formal caregivers of people with dementia.**

**Information overload**

- Risk of feeling monopolized by the system
- (Un)certainty of whether to respond to monitoring information
- Risk of disturbing daily work routines

**Privacy concerns**

- Risk for misuse of monitoring data
- Risk of losing control about data sharing
- Tradeoff privacy infringement versus extended independence

**Ethical concerns: dehumanizing care**

- Risk of replacing human contact by technology
- Risk of undermining the formal caregiver’s professional view

##### Information Overload

A topic of concern among informal and formal caregivers was the danger of information overload and feeling monopolized by an unobtrusive in-home monitoring system. Uncertainty about whether to respond to information would cause stress rather than reassurance. Both groups noted they do not feel the need to continuously check for new information but instead would like to be able to obtain a global picture of the patient’s situation.

For example, in response to the question “What would prevent you from using such a system with your loved one?”, one participant indicated:

If we would go completely nuts. If we have the feeling that we always have to go there. Sometimes it is nice that you don’t know everything, too. We are not always at home and when someone has to get into the car first…

caregiving daughter, age 58

Formal caregivers generally found it less of a problem to obtain more information than usual as they are experienced at setting priorities when responding to care needs. Some formal caregivers were rather concerned that receiving information from an unobtrusive in-home monitoring system could add an extra burden as their work might become less plannable, thereby disturbing work routines:

It might even become a burden, unplannable care.

district nurse

##### Privacy Concerns

Both formal and informal caregivers saw misuse of monitoring data as a risk for the patient, and pleaded for strict protection mechanisms during collection, processing, and sharing of data. Several formal caregivers considered the degree of privacy invasion by an in-home monitoring system to be comparable to the current professional electronic patient records, which informal caregivers can also read in real time:

But what is the difference if you write in the report “I see madam has been sad almost all morning” or if you read in the [monitoring] system “Madam has been sad all morning”?

personal care assistant

The extent to which privacy concerns about unobtrusive in-home monitoring were perceived as a problem by caregivers was dependent on what they expected to receive in return. Several caregivers weighted their concerns against the benefit of unobtrusive monitoring. In general, caregivers were willing to accept some privacy violation of their loved one/client in exchange for more safety and reassurance, independence, and quality of life:

I think that when you can keep the quality of life at a higher level by giving your privacy a little less protection, quality of life comes first.

caregiving brother-in-law, age 45

Both groups generally were less critical about being monitored themselves as well during visits or care moments but would like the option to turn off the system at any time. Most informal caregivers were willing to share monitoring information to update formal caregivers. In turn, the formal caregivers would like to share information that is relevant for the electronic patient record within their team.

However, both groups saw a risk of losing control about data sharing and highlighted the need to maintain maximum control together with the patient as data owner:

I would like to attach very specific conditions to whom you are sharing it with.

caregiving wife, age 75

##### Ethical Concerns: Dehumanizing Care

Informal and formal caregivers expressed that an unobtrusive in-home monitoring system should not be at the expense of human contact. Both groups considered it a risk that such a system might make it easier to create distance from the patient. A caregiving brother-in-law (age 45) commented:

It is because a home care organization can very easily say “We are very busy, we see in the overview that today everything is fine with Ms. X, so we will visit her tomorrow and skip her today.” I also see a risk factor because it might become easier to create distance.

Although face-to-face contact was seen as important, several formal caregivers also noted that some patients prefer as few home visits as possible. If these patients could be monitored remotely in an unobtrusive way, it could prevent disturbance and unrest:

I sometimes have night shifts where I control if someone lays in bed, but then you also disturb someone because you enter their house and that gives a certain unrest.

case manager dementia

Formal caregivers, in particular, believed that unobtrusive in-home monitoring should be an additional resource and not a substitute for their professional view. They saw a risk that such AI-driven systems might undermine their professional identity:

I think the monitoring should always provide support and it shouldn’t be the main resource. It still has to remain human work.

personal care assistant

It [the system] should not replace the professional view. It should be purely supportive and not determining, […] of course codetermining what you do, but simply be used to improve care.

occupational therapist

### Requirements Toward Unobtrusive In-Home Monitoring

#### Overview

Table 4 presents the identified requirements for the development of unobtrusive in-home monitoring systems that aim to support home-based dementia care. The requirements mainly center around what is needed from in-home monitoring technology to realize preventive and proactive measures, and to reduce identified barriers. Separate full descriptions for each requirement, including example quotes to illustrate the data behind them, are provided in Multimedia Appendix 3.

**Table 4:** Requirements toward unobtrusive in-home monitoring based on informal caregiver (IC) and formal caregiver (FC) statements.

Themes and attributes			Requirement type^a^	Requirement		Indicated by IC		Indicated by FC
**Supporting prevention and proactive measures**								
	Active support in daily living	Functional		Voice-based coaching function	Yes		Yes	
	Safety support	Functional		Autonomously detecting emergency situations and sending alarms	Yes		Yes	
	System as analysis tool	Functional		Recognizing patterns and deviations	No		Yes	
**Preventing information overload**								
	Tailored information	User experience		Information choice option	Yes		Yes	
	Tailored information	Work context		Outgoing information tailored to professional care context	No		Yes	
	One system	Work context		Integration into existing electronic client records	No		Yes	
	Creating overview of patient’s situation	User experience		Information summaries at specific time intervals	Yes		No	
**Reducing privacy concerns**								
	Transparency/safety	Functional		Preventing unauthorized access	Yes		Yes	
	Minimized obtrusion	User experience		Unobtrusive design	Yes		Yes	
	Safe interconnected system	Functional		Secure data sharing with formal caregivers	Yes		No	
	Safe interconnected system	Functional		Secure data sharing among formal caregivers	No		Yes	
	Remain in control of data	Functional		Fine-grained data sharing options	Yes		No	
**Reducing ethical concerns (dehumanizing care)**								
	Room for professional’s view	Work context		System must be supportive, not determining	No		Yes	
	Awareness for individual context	Functional		Context-aware system	Yes		Yes	
	Support ethical use	Service		Preuse instruction for caregivers	Yes		Yes	
	Support ethical use	Service		Shared decision-making tools	Yes		Yes	

^a^Classification based on van Velsen et al [40]. Functional: requirements specifying desired technical features of the technology; User experience: requirements specifying how the technology should interact/communicate with the user; Service: requirements specifying desired services surrounding the technology; Work context: requirements specifying how the technology should be integrated into the existing work context and routines (concerning use within a formal care setting).

#### Functionality

Participants provided concrete examples of how unobtrusive in-home monitoring should go beyond monitoring by providing the patient with active support in daily living via a voice-based interface. Such a coaching function could aid in tasks that typically require learning and decision making. As described by participants, assistance should focus on maintaining day structure (eg, reminders about certain times for eating, sleeping, or medication intake) or, based on the level of inactivity, providing suggestions for exercising or taking a walk.

Furthermore, as formal caregivers are interested in deviations over time, with a special interest in detecting periods deviating from the expected disease progression, the system should enable recognizing patterns and deviations, and provide the possibility to analyze on a weekly or monthly basis, thereby functioning as an analysis tool.

Moreover, the system should be capable of autonomously detecting emergency situations and sending alarms to the most suitable caregiver(s) in charge, thereby resolving the problem of current (mostly wearable) safety technology that the patient might forget to wear or might be out of reach in emergency situations. To prevent false alarms, the system should open a communication channel to the patient first to check the need to intervene. However, the system should generally be context-aware instead of applying generic threshold values in determining alarming deviations.

Furthermore, as indicated by participants, the system must offer fine-grained data-sharing options for sharing care-relevant information with formal caregivers, thereby requiring a secure connection to existing electronic client records used in professional home care. Provided that consent of the patient and informal caregiver(s) is given, the system should offer possibilities for data sharing among the professional care team as well. Communication within such a system between home care professionals and therapists or the general practitioner is seen as a desirable function to simplify multidisciplinary collaboration, but requires interoperability.

Lastly, the system must prevent unauthorized access during collection, storage, and sharing of data, thereby highlighting the importance of carefully making choices such as local vs cloud-based data processing.

#### User Experience

In line with participants’ answers, the system must provide choice options for (types of) outgoing monitoring information and its frequency of delivery to avoid information overload. For nonacute aspects, the system should provide information summaries created at specific time intervals determined by the caregiver (eg, once/twice per week, biweekly). The information needs to be summarized in a way that allows caregivers to intuitively understand the situation and make judgments on how to respond. In most cases, summarizing all information to be able to review the week is seen as sufficient, as creating a sense of overview is key.

Furthermore, the level of unobtrusiveness of the system should be maximized. Key attributes of unobtrusiveness as expressed by participants include: (1) contactlessness, being passively guarded by the system in a low-effort manner and without having to wear devices or demanding active engagement; (2) simplicity, making the system easy to use to minimize dependence on help from others; (3) privacy-friendliness by solely monitoring motion and sound; and (4) reduced visibility through a pervasive design (built into the environment), thereby minimizing the chance of stigmatization and feeling constantly reminded of the system.

#### Services Surrounding the Technology

Participants’ statements clearly showed that certain knowledge is required to prepare (in)formal caregivers for using unobtrusive in-home monitoring. We found that not only instructions on technical aspects of use are needed but, above all, instructions surrounding the ways of interacting with the patient while using the system are necessary. This is due to the fact that such systems were seen as likely to affect the caregiver-patient relationship and the amount of human contact with the patient. As indicated by the participants, one danger represents the development of a confrontational attitude when addressing monitoring information to the patient, which creates resistance. Instead, a respectful attitude directed at stimulating the positive is preferred.

Furthermore, participants indicated that the system must be introduced in a way that enables patients and (in)formal caregivers to make informed decisions based on realistic benefits and risks, thereby highlighting the need for shared decision-making tools. To prevent undermining the patient’s autonomy in case they can no longer express their will reliably, a patient declaration from the previous, competent period should be used.

#### Integration to an Existing Work Context

Formal caregivers expressed that the system must be integrated into existing electronic client records to avoid having to collect care-relevant information from different information sources/systems, which will require interoperability. Outgoing monitoring information should be tailored to the professional care context, which means that it is presented in a way that matches the content structure of the existing client record (eg, the client care plan and care goals). Mail notifications should be sent in case new monitoring summaries have been added to the client record.

Moreover, formal caregivers highlighted that to reach a fit with existing work routines and prevent undermining their professional identity, an AI-driven system must be supportive and not determining. It must therefore unlock the monitoring data in a way that care professionals can draw adequate conclusions themselves. The system can help to make the picture more complete; however, it must leave room for the care professional’s interpretation, thereby functioning as an extra aid to improve the professional view and quality of care.

## Discussion

### Principal Findings

The aim of this study was to explore the expected benefits, barriers, and relevant monitoring goals toward unobtrusive in-home monitoring from the viewpoint of formal and informal caregivers of community-dwelling people with dementia. Specific requirements that can guide the development of unobtrusive in-home monitoring technology were extracted. Extending previous work of others in the field that mainly included the views of healthy older adults [25-28,42], our study contributes to a better understanding of formal and informal caregivers’ expectations referring to the newest generation of AI-driven in-home monitoring systems, and what is needed from such systems to support home-based dementia care in an unobtrusive way. In that regard, our study provides a response to previous research that highlights the need for concrete requirements of monitoring systems in home-based dementia care [13], and argues that the purpose of such technologies needs to be regularly reviewed to keep up with their rapid development and the changing needs of users [43]. 

In general, we found that both formal and informal caregivers of people with dementia saw unobtrusive in-home monitoring as a support tool that could contribute to a shift from reactive to more preventive and proactive care. Both groups expected such systems to inform about, above all, (the risk of) falls, day and night rhythm, personal hygiene, nocturnal restlessness, and eating and drinking behavior, suggesting that these systems could best be used for people with dementia at risk for self-neglect. Although both groups showed comparable monitoring preferences, informal caregivers appeared to be less interested in monitoring eating and drinking behavior, nocturnal restlessness, and walking distance/speed compared to formal caregivers. These monitoring goals might have been less relevant to informal caregivers living together with their care recipient (36.8%), who might already have adequate supervision for these daily activities. Our findings contribute practically to gaining a better understanding of the information needs of (in)formal caregivers of people with dementia toward AI-driven in-home monitoring systems. However, in line with Elers et al [42], we generally recommend that people with dementia should always be in control of what information is collected.

Our results revealed that formal and informal caregivers of people with dementia generally expected cross-checking self-care information, extended independent living, objective communication, prevention and proactive measures, reassurance, and personalized and optimized care as the key benefits of unobtrusive in-home monitoring. At the same time, main concerns centered around information overload, privacy, and ethics.

With our focus on AI-driven in-home monitoring systems, our findings update those of Zwierenberg et al [10], who studied expectations of using rather simple monitoring systems tracking the location and movement of people with dementia. Interestingly, our study found that some benefits and barriers were two-sided, meaning that in some situations a barrier could even become a benefit and vice versa. This provided insight into novel opportunities and challenges for unobtrusive monitoring in home-based dementia care that yield implications for using such systems in a more targeted manner.

In line with previous research [31,44-46], we found that both formal and informal caregivers were concerned about consequences related to replacing human contact by technology. However, in contrast to earlier research [31,44-46], some of our participants indicated that less face-to-face contact does not always need to be a concern but might even become a benefit in certain situations. We found that in-person visits are frequently performed only as a means to control self-care behaviors of people with dementia, such as eating, drinking, and sleeping. At the same time, formal caregivers mentioned patients that prefer as few home visits as possible to prevent disturbance and unrest. Our findings show that using unobtrusive in-home monitoring from a distance might help to replace obtrusive and undesired control visits, thereby saving the caregiver’s time while supporting the patient’s sense of (physical) privacy. In that way, unobtrusive in-home monitoring may contribute to the better utilization of resources in home care, now and even more in the future.

Previous research among healthy older adults and their informal caregivers [25,47] showed that in-home monitoring systems are expected to enable extended independent living. This expectation was not fully shared by all of our participants. It became clear that formal and informal caregivers generally expected unobtrusive in-home monitoring to help prevent health risks. However, whether this proactive care would lead to extended independent living of people with dementia was not always clear to our participants. Most (in)formal caregivers expected the system to help delay institutionalization, whereas others hoped it would help to more quickly recognize when living at home would no longer be a reasonable option. The development of unobtrusive in-home monitoring technologies should therefore not be purely justified based on their potential to prolong independent living but rather based on their potential to deliver home-based care in a more beneficial way for both the patient and caregiver.

Our findings showed that unobtrusive in-home monitoring is not only seen as a technical innovation but also as a care process innovation. The last two-sided theme that emerged centered around personalized and optimized care. Formal caregivers expected that unobtrusive in-home monitoring would help them to work in a more person-centered way by tailoring care moments to the individual rhythm of the patient. However, in contrast to earlier research among formal caregivers of people with dementia [10], some of our participants expressed a tension between the need to deliver just-in-time/spontaneous care moments based on the monitoring data and work becoming less plannable as a consequence. Our findings indicate that the use of unobtrusive in-home monitoring asks for a shift in the way in which formal caregivers work from a structured to more flexible practice. We recommend considering this as an essential determinant when it comes to implementation.

In connection to the ongoing debate about privacy, our findings showed that participants were concerned about informational privacy (eg, misuse of monitoring data) and, to a lesser extent, physical privacy (eg, ‘’being monitored’’). However, the extent to which these privacy concerns were perceived as problematic by (in)formal caregivers depended on the degree of safety, reassurance, and quality of life they expected to receive by the system in return. This “trade-off” phenomenon has been recognized in earlier research [10,25]. Although privacy intrusions were principally seen as justifiable by our participants, this does not resolve possible moral considerations involved in the use of remote monitoring systems for people with dementia [10]. For instance, future developers of AI-driven in-home monitoring systems should address the need for fairness, accountability, and transparency of algorithms [48].

The requirements identified in this study recommend several ways in which unobtrusive in-home monitoring of people with dementia should be designed in terms of functionality, user experience, accompanying services, and integration into the existing work context to enhance future acceptability. Part of our requirements are in agreement with previous research related to the broader category of technology to assist aging in place [42], which highlighted the need for detection of deviations, asking minimal action from the user, and secure information storage and transfer. Furthermore, our study generated several novel requirements that centered around proactive measures, and ways to reduce barriers relating to information overload, privacy, and ethics. In the following, we address three requirements that should be given more attention.

First, our participants provided concrete examples of how unobtrusive in-home monitoring technology should be enriched with functions providing patients with active support in daily living and maintenance of day structure via a voice-based interface. Participants spontaneously came up with these ideas, which was unexpected in some ways, as the interview and focus group guide did not address these topics. This highlights that there is a true need for in-home monitoring technology to go beyond safeguarding people with dementia, and further assist in reaching personalized goals and executing tasks independently.

Second, we found that to prevent undermining the formal caregiver’s professional identity, unobtrusive in-home monitoring must be supportive and not determining. These technologies should therefore be carefully introduced and any misconceptions relating to being controlled by the technology should be corrected. Our formal caregivers expressed that the system must unlock the monitoring data so that they can draw adequate conclusions themselves. This raises questions about an optimal ratio between human and algorithmic interpretation of the data. Based on our results, we recommend more processed and simplified data for the informal caregiver and less processed data for the formal caregiver.

Lastly, an important requirement for unobtrusive in-home monitoring systems is the integration into existing electronic client records to avoid formal caregivers having to collect care-relevant information from different sources/systems. Possibilities for secure data sharing between members of the formal care team (eg, home care professionals, therapists, general practitioners) were seen as a desirable function to support integrated dementia care. Although integrated care can improve the quality of care [49], it requires interoperable information systems. This becomes even more true when care providers from different agencies, informal caregivers, and patients are involved. Interoperability issues therefore first need to be solved to enable the optimal integration of in-home monitoring systems for people with dementia into the health care system.

### Strengths and Limitations

Through in-depth conversations with various types of informal caregivers across a broad range of living situations and formal caregivers from 7 different care institutions, we were able to obtain rich information until the point of saturation was reached, which can be considered a strength of our study. Previous research noted that participants of pilot studies in particular might be more likely to have a positive orientation toward the technology, leading to bias [10,50]. Our study on expectations and needs with nonusers of unobtrusive in-home monitoring might have overcome this issue by increasing the chance of including participants that might have felt too critical or unmotivated to take part in a pilot study. In this way, our study might have been successful in including a broader range of views. However, the fact that actual use was not studied comes with a downside. As indicated by previous research [28,51,52], actual use of in-home monitoring may affect how users think about these technologies and may cause the attitudes of users to change, even after a short period of use. Most research on expectations and needs toward passive remote monitoring of people with dementia, including our own work, has not yet been tested against using these technologies in daily life. However, we have tried to overcome possible difficulties to imagine the technology in question by presenting participants with examples of unobtrusive in-home monitoring. These scenarios helped them to conceptualize the idea while at the same time being able to think beyond it.

Furthermore, our study only indirectly produced information about the perspective of people with dementia via their informal and formal caregivers, whereas it would have been preferable to include people with dementia more directly. We did so during the first interview but recognized that informal caregivers felt inhibited to freely speak about their needs in the presence of the patient. Furthermore, we generally aim to involve participants in a way that prevents participant burden and at a point in technology development that suits best their capacity. A recent review by Suijkerbuijk et al [53] on the development of assistive technologies for people with dementia showed that the more concrete the research materials are, the easier it potentially becomes for people with dementia to articulate their views. We therefore believe that collecting the views of people with dementia on more tangible prototypes/concepts in subsequent steps of development might be more adequate than asking them to envision hypothetical scenarios.

Lastly, although the primary goal of this study was to gain in-depth qualitative insights, questions remain as to whether the identified benefits, barriers, monitoring goals, and requirements can be generalized to a larger sample of caregivers and other relevant stakeholders. Adding large-scale quantitative data using an integrated mixed methods approach could help to answer this question.

### Future Research

An essential component to developing acceptable in-home monitoring technologies that support home-based dementia care will be to incorporate these requirements into the design that will provide the greatest benefit for users. We plan to share our findings with involved participants to provide room for critical feedback. In addition, future research among (in)formal caregivers on actual use is needed to determine how their expectations translate into actual experiences evolving over time and personal outcomes such as delaying institutionalization or reducing caregiver strain.

Although informal caregivers of people with dementia providing care from a distance are most likely to benefit from unobtrusive in-home monitoring, this does not mean that caregivers living with their loved one cannot also benefit from this technology. Inclusion criteria for our study were intentionally inclusive for informal caregivers from different living situations. The benefit of regaining mobility and freedom that emerged in our study was mainly brought forward by informal caregivers who lived with their care recipient, indicating that there might be more possible differences in expectations related to the use of unobtrusive in-home monitoring between informal caregivers. Future research should investigate differences in needs for different informal care scenarios to create more personalized requirements for unobtrusive in-home monitoring.

Lastly, as grounded in our results, future research should investigate ways to combine unobtrusive monitoring with ways to support people with dementia in daily living and maintaining daily structure and health. Our participants provided the idea of a voice-based interface that may give reminders on certain times for eating, sleeping, or medication intake, or, based on the level of inactivity, provides suggestions for taking a walk. As commercially available, low-cost voice interfaces such as Google Home and Siri—all of which are already integrated into the lives of many—rapidly improve the ability to understand, and even anticipate, the needs of users, unobtrusive patient assistance could become more effective [18].

### Conclusions

Unobtrusive monitoring technologies that aim to provide support in home-based dementia care are developing rapidly. Our results showed that formal and informal caregivers of people with dementia shared similar perspectives and needs. Both groups generally saw value in unobtrusive in-home monitoring, and felt that these systems could contribute to a shift from reactive to more proactive and less obtrusive care. Various concerns related to privacy, ethics, and information overload have to be considered as they are likely to hinder acceptance. This study also highlights the importance of developing and introducing AI-driven monitoring systems in a way that prevents caregivers from feeling undervalued. Our requirements can inform the development of more acceptable and goal-directed in-home monitoring technologies to support home-based dementia care.

## Appendix

Multimedia Appendix 1 Explanation of unobtrusive in-home monitoring used in interviews and focus groups.

Multimedia Appendix 2 Interview and focus group guide.

Multimedia Appendix 3 Requirements for unobtrusive in-home monitoring: full descriptions and illustrative quotes.

## Abbreviations

AI: artificial intelligence
MCI: mild cognitive impairment
