# Supplementary material for: Requirements for Unobtrusive Monitoring to Support Home-Based Dementia Care: Qualitative Study Among Formal and Informal Caregivers
Source: JMIR Aging. 2021 Apr 12;4(2):e26875. doi: 10.2196/26875 (PMC8076981; doi:10.2196/26875)
Supplement: Multimedia Appendix 1 [file aging_v4i2e26875_app1.docx]

**APPENDIX 1: Explanation of unobtrusive in-home monitoring given during interviews and focus groups**

*Imagine the following:*

*The monitoring system provides information about the daily living pattern, behavior and mood of a resident through a motion- and acoustic sensor in a central location of the room/ the appartment. These automatically track and classify only the aspects that are relevant to you and your loved one/ care recipient, such as, e.g., the resident´s activities, behaviors and mood, which can be visualized on a digital platform in real-time. The system uses artificial intelligence which means it is a self-learning system which can learn to recognize daily living patterns over time. Alarms are possible if, e.g., the resident shows no activity for longer time, or any other self-set alarm. The monitoring system continuously works on the background, in a contactless manner, meaning that residents do not have to wear any devices. Within the care network, three potential recipients of the information exist: 1) informal caregivers of the person with dementia, 2) healthcare professionals involved in the care network and 3) the person with dementia him/her self.*

*
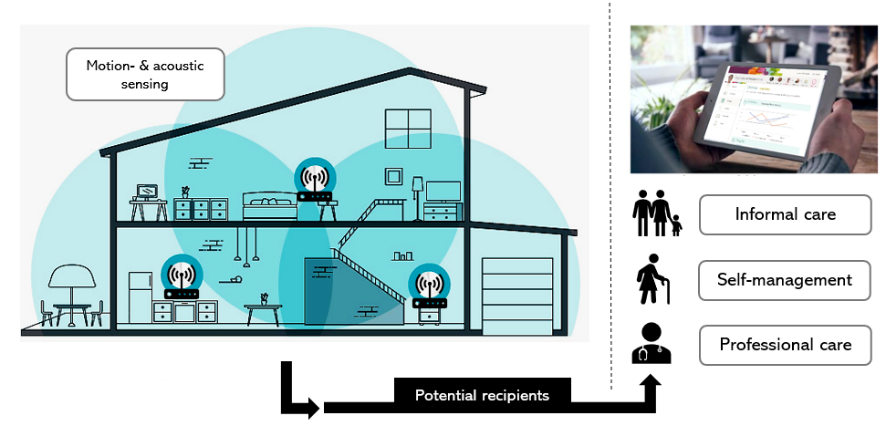
*

Slide shown to demonstrate the concept of unobtrusive in-home

monitoring. Note: Permission to use parts of the figure was granted by [tp-link.com](https://www.tp-link.com/nl/).


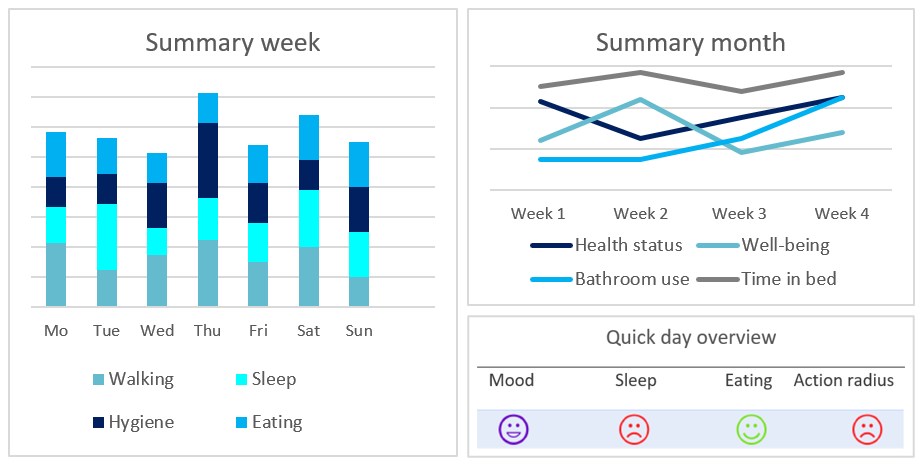


Slide shown to illustrate different examples of outgoing monitoring information
