# Supplementary material for: Requirements for Unobtrusive Monitoring to Support Home-Based Dementia Care: Qualitative Study Among Formal and Informal Caregivers
Source: JMIR Aging. 2021 Apr 12;4(2):e26875. doi: 10.2196/26875 (PMC8076981; doi:10.2196/26875)
Supplement: Multimedia Appendix 2 [file aging_v4i2e26875_app2.pdf]

APPENDIX 2: Interview- and focus group guide

Topic guide used with informal caregivers

Topic	Questions/ instructions
**Start**	Discuss with participant:<br>- Introduction of researcher and overarching project<br>- Purpose, content and structure of interview<br>- No right or wrong answers; participants are free to skip questions they prefer to not answer<br>- Participant can ask questions at any time<br>- Audio recording interview<br>- Written informed consent (anonymous processing of data, data protection measures, the right to drop out at any time)<br>- Do you have any questions so far?
**Background participant**	1. What is your age?<br>2. Do you have children?<br>a. No<br>b. Yes, co-habiting children<br>c. Yes, children living elsewhere<br>3. How long have you been informal caregiver of your loved one?<br>4. What is your loved one's age?<br>5. Which type of dementia does your loved one have?<br>6. How long has the dementia been present/ When was an official diagnosis posed?<br>7. What is the relationship between you and your loved one?<br>a. Family member (partner, son/ daughter, grandchild...)<br>b. Neighbor<br>c. Friend<br>8. Are you living together with your loved one or is your loved one living elsewhere?<br>9. How many hours per week on average do you provide care to your loved one?<br>10. What does the care around your loved one look like? Who is involved?<br>11. How do you imagine the living situation of your loved one in the coming years?<br>a. *Outside of home environment:* What would make you decide this?<br>b. *In own home environment:*<br>i. What makes you decide this?<br>ii. What additional support would be needed to keep your loved one living at home for as long as possible?<br>iii. What are you already using? How satisfied are you with it and what else do you need?
**Introduction unobtrusive in-home monitoring**	See additional file (Multimedia Appendix 1)
**Benefits**	1. What is your first reaction to such a system?<br>2. Why would you want to use such a system with your loved one?<br>a. What do you think are the benefits?<br>b. What kind of situations are there where the use of such a monitoring

	system would be useful?<br>c. What do you think of the continuous way of monitoring?<br>3. How do you think the deployment of such as system will affect the care around your loved one?<br>a. What would the information obtained by the system mean to you?<br>b. How (if at all) do you think the system will affect your caregiver duties?<br>c. To what extend (if at all) do you think such a system can make remote care easier?<br>d. How could such a system be in line with the challenges you experience?<br>e. What requirements should such a system meet to support you in the care of your loved one?<br>4. The general objective of the system is ’’Longer at home with dementia’’. Do you think this technology can achieve this?<br>a. Why?<br>b. Why not?<br>5. Do you think that the use of these systems can increase the autonomy and independence of your loved one?<br>a. Why?<br>b. Why not?<br>6. In how far (if at all) do you think such a system can help you gain a better understanding of the behavior/ habits/ well-being of your loved one?<br>7. In how far (if at all) do you think such a system can help in better adjusting the care?<br>8. What informal caregivers often find difficult is the question ’’How should I deal with my loved one?’’ or ’’How should I approach him/her?’’<br>a. What would such a monitoring system mean for you in that regard?<br>b. What would be needed from such a system to support you in this?
**Monitoring goals**	**Topic list task**<br>In front of you, you can find a list with 16 different topics. Each of these topics represents a potential monitoring goal of the system described earlier, that you might or might not want to have monitored. Please rate each goal with either a plus sign (+), indicating that this would be a relevant monitoring goal, a minus sign (-), indicating a non-relevant monitoring goal, or a question mark (?), if you are unsure about the usefulness of a certain monitoring goal. Please imagine that all goals could technically be monitored to any useful level of precision. We will go through the topics together step for step afterwards.<br><br>Questions:<br>1. Are there any additional aspects you would like to have monitored?<br>2. In case of ’’+’’<br>- When and how would you like to be informed about this?<br>- What should the information look like and how detailed should it be?<br>3. In case of ’’-‘’<br>- What makes you prefer not to be informed about this?<br>4. In case of ’’?’’<br>- What makes you doubt if you would like to be informed about this?
**Barriers**	1. What would prevent you from using such a system with your loved one?<br>a. Why would you decide not to use such a monitoring system?<br>b. What are the disadvantages/ risks in your opinion?<br>2. When would the system become undesirable?

	3. Which aspects do you see as a threat to yourself when it comes to using such a monitoring system?<br>a. Suppose you detect a worrysome change or incident via the system. How would you like to deal with this? How would this affect the extend of care burden you experience?<br>b. How do you feel about being monitored yourself by the system during moments of care or in case you are living together with your loved one?<br>4. Which aspects do you see as a threat to your loved one when it comes to using such a monitoring system?<br>a. What would you like to protect? When would you say ''Until here and not further''?<br>5. Would you like to share the information obtained from the system with others?<br>a. If yes: With whom would you like to share it (e.g. other informal caregivers, home care professionals, therapists, general practitioner)? And under what circumstances?<br>b. If not: What makes you think that? What are you afraid of?<br>6. Do you think the feeling of security is more, even or less important than guaranteeing the privacy of your loved one? Can you explain?
**End**	1. Would you like to give a final advice to developers of artificial intellingent in-home monitoring technology? If so, what would it be?<br>2. Are there any aspects that we have not yet discussed in the previous sections that you would like to share?

Topic guide used with formal caregivers

**Topic**	**Questions/ instructions**
**Start**	Discuss with participant(s):<br>- Introduction of researcher and overarching project<br>- Purpose, content and structure of interview/ focus group<br>- No right or wrong answers; participants are free to skip questions they prefer to not answer<br>- Participant can ask questions at any time<br>- Audio recording interview/ focus group<br>- Written informed consent (anonymous processing of data, data protection measures, the right to drop out at any time)<br>- Do you have any questions so far?<br>- Short get-to-know-round between participants
**Background participant(s)**	1. Background questionnaire (age, gender, years of work experience in current home care profession, care contact hours provided to community-dwelling PwD on average per week)<br>2. Do you use monitoring systems in daily practice?<br>a. If yes: Is there anything you miss about these systems?<br>b. If yes: What could they do better to support your daily work and to keep your clients living at home for as long as possible?

**Introduction unobtrusive in-home monitoring**	See additional file (Multimedia Appendix 1)
**Benefits**	1. What is your first reaction to such a system?<br>2. Why would you want to use such a system with community-dwelling clients with dementia?<br>a. What do you think are the benefits?<br>b. What kind of situations are there where the use of such a monitoring system would be useful?<br>c. What do you think of the continuous way of monitoring disease progression?<br>3. How do you think the deployment of such a system will affect the care around your clients with dementia?<br>a. What would the information obtained by the system mean to you?<br>b. How (if at all) do you think the system will affect your way of working?<br>c. To what extend (if at all) do you think such a system can make remote care easier?<br>d. How could such a system be in line with the challenges you experience?<br>4. The general objective of the system is ‘’Longer at home with dementia’’. Do you think this new technology can achieve this?<br>a. Why?<br>b. Why not?<br>5. Do you think that the use of these systems can increase the autonomy and independence of your clients with dementia?<br>a. Why?<br>b. Why not?<br>6. In how far (if at all) do you think such a system can help you gain a better understanding of the behavior/ habits/ well-being of your clients with dementia?<br>a. What do run into currently if you want to form a picture of a client with dementia? How accurate is the information you get?<br>7. How (if at all) do you think such as system will influence the quality (and adjusting) of care?<br>8. In how far do you think such a system can save you time?<br>9. What requirements should such a system meet to support you in the care of clients with dementia and accompanying the disease process?
**Monitoring goals**	**Topic list task**<br>In front of you, you can find a list with 16 different topics. Each of these topics represents a potential monitoring goal of the system described earlier, that you might or might not want to have monitored. Please rate each goal with either a plus sign (+), indicating that this would be a relevant monitoring goal, a minus sign (-), indicating a non-relevant monitoring goal, or a question mark (?), if you are unsure about the usefulness of a certain monitoring goal. Please imagine that all goals could technically be monitored to any useful level of precision. We will go through the topics together step for step afterwards.<br><br>Questions:<br>1. Are there any additional aspects you would like to have monitored?<br>2. In case of ‘’+’’<br>- When and how would you like to be informed about this?

<table>
<tr><td></td><td>- What should the information look like and how detailed should it be?
3. In case of ''-''
- What makes you prefer not to be informed about this?
4. In case of ''?''
- What makes you doubt if you would like to be informed about this?</td></tr>
<tr><td>Barriers</td><td>1. Suppose there is consent from the client with dementia and his/her family to use such a system. What would still prevent you from using it?
a. Why would you decide not to use such a monitoring system?
b. What are the disadvantages/ risks in your opinion?
2. When would the system become undesirable?
3. How should such a system communicate with you to protect your energy resources during work?
4. Which aspects do you see as a threat to yourself when it comes to using such a monitoring system?
a. Suppose you are detecting a worrysome change or incident via the system. How would you like to deal with this? How would this affect the work pressure you experience?
b. How do you feel about being monitored yourself by the system during moments of care?
5. Which aspects do you see as a threat to your clients with dementia when it comes to using such a monitoring system?
a. What would you like to protect? When would you say ''Until here and not further''?
6. Would you like to share the information obtained from the system with others?
a. If yes: With whom would you like to share it (e.g. informal caregivers, other home care professionals, therapists, general practitioner)? And under what circumstances?
b. If not: What makes you think that? What are you afraid of?
7. Do you think the feeling of security is more, even or less important than guaranteeing the privacy of your client with dementia? Can you explain?</td></tr>
<tr><td>End</td><td>1. Would you like to give a final advice to developers of artificial intelligent in-home monitoring technology? If so, what would it be?
2. Are there any aspects that we have not yet discussed in the previous sections that you would like to share?</td></tr>
</table>
