# Supplementary material for: Requirements for Unobtrusive Monitoring to Support Home-Based Dementia Care: Qualitative Study Among Formal and Informal Caregivers
Source: JMIR Aging. 2021 Apr 12;4(2):e26875. doi: 10.2196/26875 (PMC8076981; doi:10.2196/26875)
Supplement: Multimedia Appendix 3 [file aging_v4i2e26875_app3.pdf]

APPENDIX 3: Requirements for unobtrusive in-home monitoring: full descriptions and illustrative quotes

Theme	Attribute	Requirement	Type	Example quotes	Brought forward by[a]	
					IC	FC
Prevention and pro-active measures	Active support in daily living	**Voice-based coaching function**<br>The system should go beyond monitoring and provide means for assisting the patient via a voice-based interface in order to support daily living and enhance well-being, either data-driven (based on monitoring data) or determined by caregiver. As described by participants, assistance should focus on maintaining day structure (e.g. reminders on certain times for eating/ sleeping/ medication intake) or, based on the level of inactivity, providing suggestions for excercising or taking a walk.	Functional	*People with dementia often want to do things and they can make choices, but they will not always execute them. Then such a system can take over.* [caregiving daughter, 50]<br><br>*R: Okay, and do you also think that it will allow people to live at home longer?*<br>*P: Yes absolutely. It could help someone to structure, to stimulate positive behavior. The sensors can also indicate that someone might need to go to the bathroom so that they don't wet their pants.* [caregiving daughter, 50]<br><br>*What could be very nice is - and this could be an idea for the system – in the moment you notice that someone is getting restless in the pajamas, that you then give a signal ''Sir, it is time to go to bed. Good night.''* [occupational therapist]	X	X
	Safety support	**Autonomously detecting emergency situations and sending alarms**<br>The system must be capable of autonomously detecting emergency situations and sending alarms to suitable professionals/ caregivers, based on the type of emergency situation, thereby resolving the problem of current (mostly wearable) safety technology that a) might be forgotten to wear, b) is rejected by patient or c) might be out of reach in emergency situations. To prevent false alarms, the system should open a	Functional	*When it comes to falls, I actually want the healthcare professional to be called immediately. And if the healthcare professional is called, it is good if you get the right people in the right place to be able to help as well as possible.* [caregiving daughter, 42]<br><br>*Well, we would anyways like to have something developed so that you can see if someone has fallen, because you also have clients with dementia who do not know or understand how such an alarm works. So I think*	X	X

		communication channel to the patient first in order to check the need to intervene.		*that would make your job even more professional.* [district nurse]<br><br>*P: Then you can leave even more reassured because you know, if something happens, everyone is called and that is ideal. Now if something happens and he [husband with dementia] doesn't call himself, then he just lays there [on the ground]. And if he calls, then something will be done, but then the professionals such as doctors etc. will not come, you have to do all that extra.*<br>*R: So you would actually need to have this task done for you?*<br>*P: Yes, that would be very nice, because then I can go on with my own life.* [caregiving wife, 72]		
	System as analysis tool	**Recognizing patterns and deviations**<br>Loose monitoring scenarios sometimes do not provide enough information. Care professionals are interested in the bigger picture and deviations over time in order to draw meaningful conclusions and respond to them. A special interest lays in detecting periods deviating from the expected disease progression or periods of faster decline. The system must therefore provide the possibility to analyze on week- or month level and thus create overview of patterns.	Functional	*Well, I did have a client who lived alone and was reversing his day- and night rhythm, where we also had the question how does it come that he turns that around? Is he not very active during the day or is it the environment that makes him become passive? And by analyzing movement and sound you can recognize a pattern in it.* [case manager dementia]<br><br>*I think it will be more about getting a good picture about the pattern. You know, if someone doesn't wash himself for a day, that doesn't matter, you want to know when the pattern changes.* [personal care assistant]		X
Information overload	Tailored information	**Information choice option**<br>The system must provide choice options for (types of) outgoing monitoring information and the frequency of delivery of that information in order to avoid information overload, attention theft and overcharging of its users. The system must provide tailored information. Caregivers should be able to adjust the system information settings to their own needs.	User experience	*My first reaction would be that I would like to use it [the system] focused on specific persons where you then measure one, two or three aspects and not everything and that you will also do this over a certain period of time. Personally, if needed, I would like to apply it [the system] on specific aspects.* [caregiving neighbor, female, 77]	X	X

				You should be able to click on what you want information about at that moment. For example about toilet use if someone is incontinent throughout the day. That you only get that information and that you can simply have insight into the rest if you want to. [district nurse]		
	Tailored information	**Outgoing information tailored to professional care context**<br>The system must provide caregiver access to it's collected information with the possibility to filter on client and monitoring target to avoid information overload. Extensiveness and frequency of information feedback should be able to be set by patients and caregivers.<br>Acute aspects that require immediate intervention should be notified via telephone to the responsible emergency service. The system should furthermore create overviews of monitoring data about all clients as input for (monthly) multidisciplinary team meetings.	Work context	*[…] I can imagine that for one client you would be happy with a smiley in red, orange or green once a week and for another client you would like to have a daily chart on how it goes from hour to hour.* [case manager dementia]<br><br>*P: To me it seems most conventient if you, for example, receive the information from all clients that have it [the system] once per month via email.*<br>*R: Sort of an overview?*<br>*P: Yes exactly, because we also sit together with the general practitioner and other disciplines once a month, so you could discuss it like ''This and this strikes me'' or maybe someone else notices something. You can discuss that during a team meeting.* [district nurse]		X
	One system	**Integration into existing electronic client records**<br>The system must be integrated (fully or via link) into existing electronic client records/ report systems in order to avoid care professionals having to collect care-relevant information from different information sources/ systems.<br>Outgoing monitoring information should be presented in a way it matches the content structure of the existing electronic client record (e.g. the client care plan and care goals). Mail notifications should be sent in case new monitoring summaries have been added to the client record.	Work context	*[Information should be] in the report, linked to the care goal. If I want to monitor agitation for instance, or mood, and a client suddenly starts throwing cups, then I would like the system to immediately note this at the care goal.* [district nurse]<br><br>*It would help me if I make a note in the care plan that I would like to monitor the nighttime rest, that all figures or information like ''Madam has been out of bed 10 times'' immediately appear in the client report [...] Then I have all in one system, instead of having to gather information from different systems. And also immediately related to the care goals.* [district nurse]		X

	Creating overview of patient's situation	**Information summaries at specific time intervals**<br>For non-acute aspects, the system should provide data summaries created at specific time intervals determined by the caregiver (e.g. once/ twice per week, bi-weekly ect). The information needs to be summarized in a way that allows caregivers to intuitively understand the situation and make judgments on how to respond to it. In most cases, summarizing all information to be able to review the week is seen as sufficient, creating overview stays central. For acute aspects that require immediate intervention the system must provide mobile access to caregiver.	User experience	*Once a week I would be very happy to go through the whole week and see what has and what has not happened and what I can do about it myself. But if I had that [the monitoring information] every day or in the moment itself, I don't really think it would make me feel good.* [caregiving wife, 72]	X	
Privacy concerns	Transparency/ safety	**Preventing unauthorized access**<br>The system must protect data during collecting, storage and sharing, thereby avoiding misuse of data.<br>The system must provide means for and stick to clear agreements on data storage- and sharing.<br>Choices such as local vs. cloud-based processing of monitoring data should carefully be made based on a user-centered perspective.	Functional	*Actually, if it [the monitoring information] goes outside where it is not intended, then people can take the advantage and break in because they might see ''Oh it is a vulnerable elderly person and no one is at home.'' And then it may not be that safe.* [caregiving daughter, 50]<br><br>*In any case, there need to be clear agreements with the familiy, discussing privacy-sensitive information and determining how to deal with it.* [district nurse]	X	X
	Minimized obtrusion	**Unobtrusive design**<br>The level of unobtrusiveness of the system should be maximized. Key attributes of unobtrusiveness as indicated by participants include:<br>- Contactlessness: Being passively guarded by the system, demanding low effort and without having to wear devices. According to participants, contactlessness not only provides benefits in the moderate to late stages of dementia but also in the very early stages where patients often don't see a point in using wearables.<br>- Simplicity: Easy to use in order to minimalize dependence on help from others	User experience	*That [the system] is of course something that is otherwise invisible and you don't have to carry a box or install an app on your phone. It all goes automatic and it is somewhere in the house, you will get used to it. It would be ideal for me and I am sure I would calm down.* [caregiving wife, 72]<br><br>*P2: Well, what immediately appeals to me is that it is passive and that you don't have to wear anything or do things.*<br>*P5: You don't have to turn it on etc.*<br>*P2: Because you probably won't learn that anymore with dementia.* [district nurse and occupational therapist]	X	X

		- Privacy-friendliness by solely monitoring motion and sound<br>- Reduced visibility through pervasive design (built into the environment), thereby minimazing stigmatization and the chance of feeling constantly reminded of the system.		*If I may think practically, because we are not that technical, we would probably depend on others in the beginning. It must be an easy system, otherwise not everyone can use it.* [caregiving daughter, 50]<br><br>*If you have a system where, for example, the client has to press a button and something like that, that will not work to learn a new action.* [personal care assistant]		
	Safe interconnected system	**Secure data sharing with formal caregivers**<br>The system must offer an option for sharing specific care-related monitoring data with formal caregivers in a secure way, in order to update them/ consult them for advice. The system must therefore be capable of connecting to existing report system structures that are used in professional home care.	Functional	*R: So you could imagine sharing this information also with the healthcare professional?*<br>*P: Yes, for sure, that is very important to me, because you need that information to show how things are going here.* [caregiving wife, 72]<br><br>*P: I think the analysis is fine to share, but I also think that some kind of protection is needed.* [caregiving daughter, 42]	X	
	Safe interconnected system	**Secure data sharing among formal caregivers**<br>Provided that consent of patient and informal caregiver(s) is given, the system should offer possibilities for sharing specific care-relevant monitoring data in a secure way among the formal care team in order to update each other/ consult each other for advice. The system must therefore be interoperable with existing electronic client records that are used in professional home care. Communication within such a system between between care providers of different agencies (e.g. home care professionals, therapists, general practitioners) is seen as a desirable function.	Functional	*I think that for the personal assistance it can be very interesting to have indeed more background information in order to see where are the deviations, but that a summary thereof also goes to the general practitioner for example or to another therapist so that you can see together how the situation is and and where we can indeed take action. But I think don't just give everyone the information.* [occupational therapist]<br><br>*[Sharing information] with close relatives or informal caregivers, family, acquaintances, if they wish to and the client allows it. And of course with all colleagues of the team.* [district nurse]<br><br>*[…] That it [the system] handles the information confidentially, that not everyone has access to it, except from the people who are linked to it.* [district nurse]		X

	Remain in control of data	**Fine-grained data sharing options**<br>The system must provide fine-grained data sharing options to its users, tailored to their role and needs within the care network. The system must let the primary user own his/her data and remain control of data sharing at any time.	Functional	*[...] The general practitioner as a regular care coordinator, but then, I would not want to share it [the information] standardly with every care coordination because I have had bad experiences with that. I sometimes find it a problem how they deal with privacy, especially if you live in a small town. [...] I would like to attach very specific conditions to whom you are sharing it with.* [caregiving wife, 75]	X	
Ethics (Dehumanizing care)	Room for professional's view	**System must be supportive, not determining**<br>The system must unlock the monitoring data in a way that care professionals can draw adequate conclusions themselves. The system can help to make the picture more complete, however, it must leave room for the care professional's interpretation, thereby functioning as an extra aid to improve the professional view and quality of care.	Work context	*If you can use this kind of data to simply make your picture more complete, it should still be up to you as professional to interpret the data, how you indeed address it and what you then do with it in your daily care provision.* [occupational therapist]<br><br>*I think the monitoring should always provide support and it shouldn't be the main resource. It still has to remain human work.* [personal care assistant]		X
	Awareness for individual context	**Context-aware system**<br>The system must be context-aware and algorithms should be trained per person. Each patient lives within an individual situation. Recognition of behavioural patterns and deviations is desirable but the system should not use generic threshold values. Moreover, taking contextual factors into account has the potential to provide insight into the causes of misunderstood behaviour and emotion.	Functional	*It [the system] should not be an automatic pilot, like one plus one is two, but you have to look between those one and two, could it also be one and a half? Could it also be different?* [caregiving daughter, 50]<br><br>*People with dementia often have misunderstood behavior [...] Suppose there is a stimulus that causes someone to have night-time unrest or to wander. Normally, wandering is a criterion that prevents someone from living at home. Suppose you can remove this stimulus because you know where it comes from, then you ensure that someone can stay home longer. And in that sense I think the system is an added value.* [case manager dementia]	X	X
	Support ethical use	**Pre-use instruction for caregivers**	Service	*The danger is that the family is in control of everything. And especially in the case of dementia, when people*	X	X

		The system was seen as likely to affect the caregiver-patient relationship and the amount of human contact with the patient. To prepare informal and formal caregivers for using the system not only instructions on technical aspects of use are needed but especially instructions surrounding the ways of interaction with the patient while using the system. One danger represents the development of a confrontational attitude when addressing monitoring information to the patient which creates resistance. Instead, a respectful attitude directed at stimulating the positive is preferred.		*[caregivers] emphasize so often what you can not do, it only produces resistance. I think that information for the family about how to deal with dementia is very important before you start with such a system.* [personal care assistant]<br><br>*You can say ''I have seen this and this.'' But you can also say ''Would it seem fun or pleasant to you if you...'', that you address it [the monitoring information] in a completely different way.* [case manager dementia]		
	Support ethical use	**Shared decision-making tool**<br>The system must be introduced in a way patients and (in)formal caregivers can make informed decisions based on realistic benefits and risks. The patient's will must be central. If patient cannot express his/her will reliably anymore, a patient declaration from the previous, competent period, should be used.	Service	*You are still at the starting point with this kind of technology and I hope that it will be developed further in the long-term and that people can explain in their own client record what they want and what they don't want, so that they are themselves in conrol of that. [caregiving daughter, 42]*<br><br>*P2: As soon as someone is no longer capable of doing this [providing consent], the family may decide.*<br>*P4: But still then I think if someone has said ''No, no, no'' all his life [...] and then the children are allowed to decide, then I still wonder to what extend I agree.*<br>[personal care assistant and case manager dementia]	X	X

[a]IC= Informal caregivers; FC= Formal caregivers

[This is a Multimedia Appendix to a full manuscript published in JMIR Aging. For full citation information see http://dx.doi.org/10.2196/26875.]
